# Effect of hemodialysis on extracellular vesicles and circulating submicron particles

**DOI:** 10.1186/s12882-019-1459-y

**Published:** 2019-08-02

**Authors:** Marcel Ruzicka, Fengxia Xiao, Hussein Abujrad, Yasamin Al-Rewashdy, Vera A. Tang, Marc-André Langlois, Alexander Sorisky, Teik Chye Ooi, Dylan Burger

**Affiliations:** 1 Kidney Research Centre, Ottawa Hospital Research Institute, University of Ottawa, 2513-451 Smyth Road, Ottawa, Ontario K1H 8M5 Canada; 2 Division of Nephrology, University of Ottawa, The Ottawa Hospital, Riverside Campus, Room 5-21, Riverside 1967, Ottawa, Ontario K1H 7W9 Canada; 3 Division of Cardiology, University of Ottawa Heart Institute, Ottawa, Ontario Canada; 4 Division of Endocrinology and Metabolism, University of Ottawa, Ottawa Hospital Research Institute, Ottawa, Ontario Canada; 5 uOttawa Flow Cytometry & Virometry Core Facility, Ottawa, Ontario Canada; 6 Department of Biochemistry, Microbiology and Immunology, University of Ottawa, Ottawa, Ontario Canada; 7 Department of Cellular and Molecular Medicine, University of Ottawa, Ottawa, Ontario Canada

**Keywords:** Hemodialysis, Microparticle, Extracellular vesicle, Kidney, Endothelium, Platelet, Leukocyte, Dialysis, End stage kidney disease

## Abstract

**Background:**

Although hemodialysis is a highly effective treatment for diffusive clearance of low molecular weight uremic toxins, its effect on circulating extracellular vesicles and submicron particles is less clear. The purpose of this study was to examine the impact of hemodialysis on circulating levels of submicron particles.

**Methods:**

Plasma samples from patients were collected immediately before and after the mid-week hemodialysis session. Total submicron particles were assessed by nanoparticle tracking analysis and levels of endothelial (CD144^+^), platelet (CD41^+^), leukocyte (CD45^+^), and total (Annexin V^+^) membrane microparticles (MPs) were assessed by flow cytometry.

**Results:**

Total submicron particle number was significantly lower post-dialysis with reductions in particles < 40 nm, 40–100 nm, and 100–1000 nm in size. Circulating annexin V^+^ MPs, platelet MPs, leukocyte MPs, and endothelial MPs were all reduced following dialysis. Assessment of protein markers suggested that extracellular vesicles were not present in the dialysate, but rather adsorbed to the dialysis membrane.

**Conclusions:**

In summary, hemodialysis is associated with reductions in circulating submicron particles including membrane MPs. Accordingly, there may be significant interdialytic variation in circulating submicron particles. Investigators interested in measuring extracellular vesicles in patients undergoing hemodialysis should therefore carefully consider the timing of biosampling.

## Background

Human blood contains a variety of submicron entities including lipoproteins, viruses, protein complexes, and extracellular vesicles (EVs). EVs are membrane-enclosed vesicles that are formed by all cells under physiological and pathophysiological conditions. EVs may be further subdivided into small EVs (~ 40–100 nm, often referred to as exosomes) and large EVs (100–1000 nm, often referred to as microparticles, MPs). Although most studies do not directly assess the biogenesis of EVs, exosomes are conventionally described as arising from endosomal sorting processes and extracellular release following fusion of multivesicular bodies with the plasma membrane. By contrast, membrane MPs are typically defined as originating from outward membrane blebbing under conditions of cell stress/injury. In the context of chronic kidney disease circulating MPs arising from platelets, endothelial cells, leukocytes, or erythrocytes are increased and levels have been shown to correlate with measures of vascular injury [1–8]. In fact, high levels of circulating endothelial MPs is an independent predictor of cardiovascular morbidity and mortality in patients with end stage kidney disease (ESKD) [9]. Interestingly, although urinary exosomes have been extensively studied in chronic kidney disease (reviewed in [10]), information regarding levels of circulating small EVs in this condition is lacking.

In individuals with ESKD, hemodialysis is the primary modality used for diffusive clearance of low molecular weight uremic toxins. Hemodialysis is highly effective in clearing small, water-soluble compounds, increasingly efficient with middle molecular weight molecules, but less efficient in the removal of protein-bound uremic toxins [11]. To date, the effects of hemodialysis on circulating EVs (which are much larger than the pore size of dialysis filters) remains controversial. On one hand, a handful of studies have suggested that dialysis increases certain populations of circulating MPs, possibly due to the higher hemodynamic stress during the treatment [4, 5, 12, 13]. By contrast, other studies have shown little effect on circulating MPs [3, 8] and still others have shown reductions post-dialysis [14]. Clinical continuous venous hemofiltration has also been shown to reduce levels of endothelial-derived MPs [15]. Accordingly, the impact of dialysis on circulating MPs remains unclear. In addition, to the best of our knowledge, no studies have been conducted that assess the effect of hemodialysis on levels of circulating small EVs and other particles < 100 nm in size.

The purpose of the present study was to assess the effect of hemodialysis on levels of circulating EVs and submicron particles. We examined pre- and post-dialysis levels of circulating platelet, endothelial, and leucocyte MPs by flow cytometry. In addition, we examined levels of circulating submicron particles by nanoparticle tracking analysis. Relationships with ultrafiltration rate were explored. Finally, we assessed whether circulating EVs are filtered through or adsorb to the dialysis membrane.

## Methods

### Patients

Two separate cohorts of patients undergoing three times weekly hemodialysis with Fresenius FX-800 filters were studied. The FX-800 filter uses a polysulfone-based membrane for filtration. The first cohort (30 males and 26 females) was used to assess levels of circulating MPs and submicron particles pre- and post- hemodialysis treatment. Demographics of these patients are shown in Table 1. Cohort 2 (3 males and 3 females) was used for assessment of vesicle protein in dialysate and vesicle adherence to the dialysis membrane. Demographics for cohort 2 are shown in Table 2.

**Table 1:** Baseline Characteristics of Cohort 1

	Patients (*n* = 56)
Age at collection (years)	61.5 ± 2.41
Male gender (%)	53.6
Duration of hemodialysis (months)	48.0 ± 5.49
Ultrafiltration on day of collection (ml/kg/h)	7.35 ± 0.72
Weight (post-dialysis)	72.1 ± 2.6
Co-morbidities (%)	
Diabetes	41.1
Hypertension	66.1
Rheumatoid arthritis	16.1
Chronic obstructive pulmonary disease (COPD)	7.1
Coronary artery disease (CAD)/ Peripheral vascular disease (PVD)	25.0
Cancer history	16.1

**Table 2:** Baseline Characteristics of Cohort 2

	Patients (*n* = 6)
Age at collection (years)	60 ± 4.9
Male gender (%)	50
Duration of hemodialysis (months)	74 ± 26.1

### Ethics, consent and permissions

Informed written consent was obtained from all patients, and all studies were approved by the Ottawa Health Science Network Research Ethics Board (protocol #2011793-01H).

### Sample collection

Blood samples for both cohorts were collected into citrated tubes immediately before and after hemodialysis at the mid-week session. Dialysate was collected after 1 h and at the end of the dialysis session (4 h). Platelet-poor plasma was prepared by centrifugation at 2500 g for 10 min and frozen at − 80 °C until sample analysis.

### Characterization of submicron particles by nanoparticle tracking analysis

Sizing and quantification of extracellular vesicles and submicron particles in cohort 1 was achieved by nanoparticle tracking analysis (NTA) using the ZetaView PMX110 (Particle Metrix, Meerbusch, Germany) in size mode as we have done previously [16–18]. Aliquots of plasma samples were diluted in PBS to a particle concentration within the working range of the system. Approximately 1 ml of sample was loaded into the sample chamber after calibration using 105 nm and 500 nm polystyrene beads. ZetaView software (version 8.02.28) was used for analysis at 11 camera positions with a 2 s video lengths, a camera frame rate of 30 fps, and system temperature of ~ 21°C.

### Microparticle isolation and characterization

MPs were isolated from aliquots of platelet-poor plasma of patients in cohort 1 as described previously [19, 20]. Briefly, samples were thawed rapidly and centrifuged at 12,000 g for 2 min. The supernatant was then transferred to a fresh tube and centrifuged at 20,000×g for 20 min at 4 °C. The MP-containing pellet was collected, and the supernatant, containing exosomes, smaller vesicles, and soluble factors, was discarded. The MP-containing pellet was re-suspended in Annexin V binding buffer containing 10 mM HEPES, pH 7.4, 140 mM NaCl, 2.5 mM CaCl2.

Samples were labeled with 2.0 μg/ml of FITC-conjugated Annexin V (1:50 dilution, Biolegend) and either 0.25 μg/ml of APC-conjugated CD41 antibody (1:100 dilution, Biolegend), 2.0 μg/ml of PE-labeled CD144 antibody (1:100 dilution, BioLegend), or 1.0 μg/ml of BV421-conjugated CD45 (1:25 dilution, BioLegend). These concentrations were titrated to determine optimal labeling conditions. As negative controls, a sub-population of MPs was resuspended in Annexin V binding buffer lacking calcium, which is necessary for Annexin V binding to phosphatidylserine, and populations of MPs were labeled with matched fluorophore-conjugated IgG isotype controls. Label antibodies in buffer alone (no sample) were also assessed and background events were subtracted from enumerated samples to eliminate the possibility of false positives due to antibody aggregates.

Samples were analyzed at the University of Ottawa Flow Cytometry Core Facility using a custom Special Order Research Project BD LSRFortessa system. The system configuration and calibration for small particles have been described previously [18]. MPs were defined as particles between ~ 100–1000 nm in size that exhibited significantly more AnnexinV fluorescence than their negative controls. Results are number of annexinV+ (Total), annexin V+, CD41+ (platelet), annexin V+, CD45+ (leucocyte), or annexin V+, CD144+ (endothelial) MPs/ mL plasma.

### Collection of dialysate solution

To determine the fate of extracellular vesicles in dialysis we examined the presence of EV-associated protein in the dialysate of patients in cohort 2. A 50 ml aliquot of used dialysate solution was collected from the sampling port at the end of a 4-h hemodialysis session. Dialyzed samples were concentrated using a 30 kDa cutoff filter (Amicon) and the proteins were re-suspended in 50 μl of radioimmunoprecipitation assay buffer (RIPA buffer; 50 mM Tris-HCl pH 7.2, 150 mM NaCl, 1% NP40, 0.1% sodium dodecyl sulfate, 0.5% deoxycholic acid, 1 mM phenylmethanesulfonyl fluoride, 25 mM MgCl2).

### Assessment of vesicle adsorption to dialysis membrane

To determine if vesicles adsorb to the dialysis membrane, dialyzers from patients in cohort 2 were collected post-dialysis and disassembled. The interior hollow fibre membrane was cut into ~ 1 cm^2^ pieces and suspended in RIPA buffer for 4 h at 4°C. The membrane was subsequently scraped with a rubber policeman and lysates of adsorbed protein collected.

### Immunoblot analysis

40 μl of concentrated dialysate solution and/or 20 μg of adsorbed protein lysate were loaded onto 10% polyacrylamide gels and levels of the vesicle-associated protein flotillin (Santa Cruz Biotechnology) were assessed by immunoblot analysis as described previously [21].

### Statistical analysis

Data are presented visually as box plots, as the median, 25th and 75th percentiles. Statistical analysis was performed by the Signed Rank test for paired pre- and post-hemodialysis samples to compare data. Spearman’s rank-order Correlation Coefficient was calculated to identify correlation between particle clearance and particle size, and between clearance of MP subpopulations and ultrafiltration rate. Data were analyzed using SigmaStat **(**version 3.5 SYSTAT). For all data, a *p* value < 0.05 was considered significant.

## Results

### Assessment of submicron particles by nanoparticle tracking analysis

**Fig. 1:**
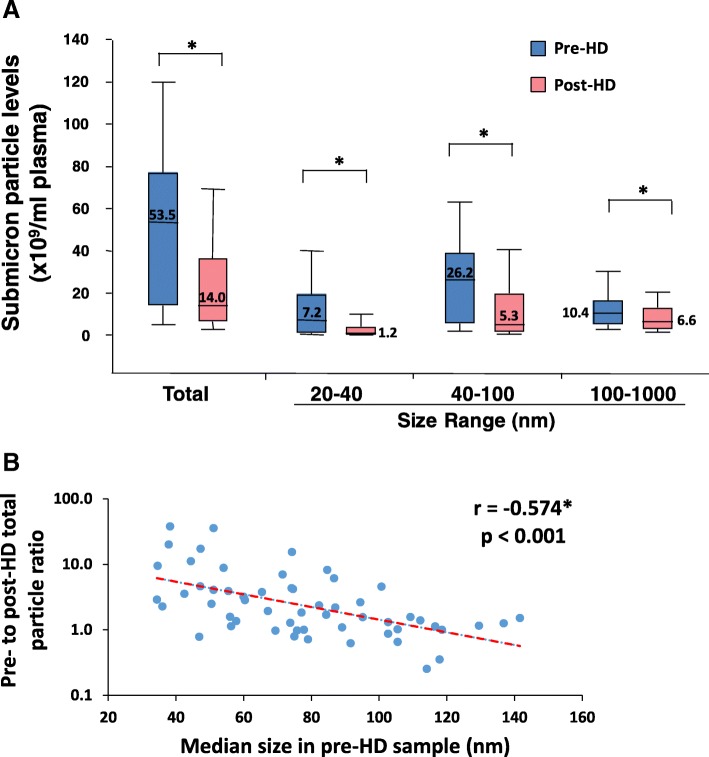
**a**: Nanoparticle tracking analysis of submicron particle levels in plasma before (pre) and after (post) hemodialysis (HD). Shown are total particle numbers for a given size range (top panel). *p < 0.001. **b**: Correlation between particle clearance (expressed as pre/post particle ratio) and pre-HD particle size (median in nm)

**Fig. 2:**
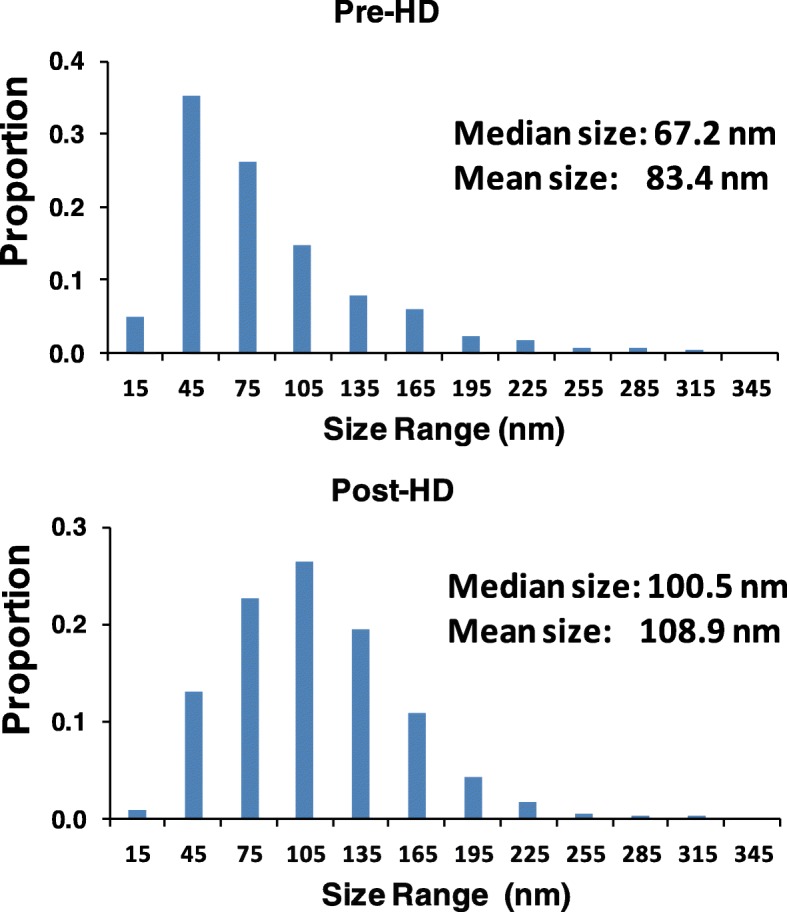
Representative size profiles from nanoparticle tracking analysis of pre and post-dialysis plasma

In order to assess the effect of hemodialysis on extracellular vesicles and submicron particles, we examined pre and post-dialysis plasma samples by nanoparticle tracking analysis. As shown in Fig. 1 the concentration of particles 20–1000 nm in size was reduced in post-hemodialysis samples. This effect was observed regardless of the particle size (Fig. 1a). The greatest reductions were seen in the smallest population (20–40 nm vs 40–100 nm vs 100–1000 nm) and an inverse relationship between particle size and clearance during hemodialysis was seen (Fig. 1b). Consistent with this, post-dialysis particles were larger than those seen prior to hemodialysis (Fig. 2).

### Effects of hemodialysis on circulating large EVs/microparticles

**Fig. 3:**
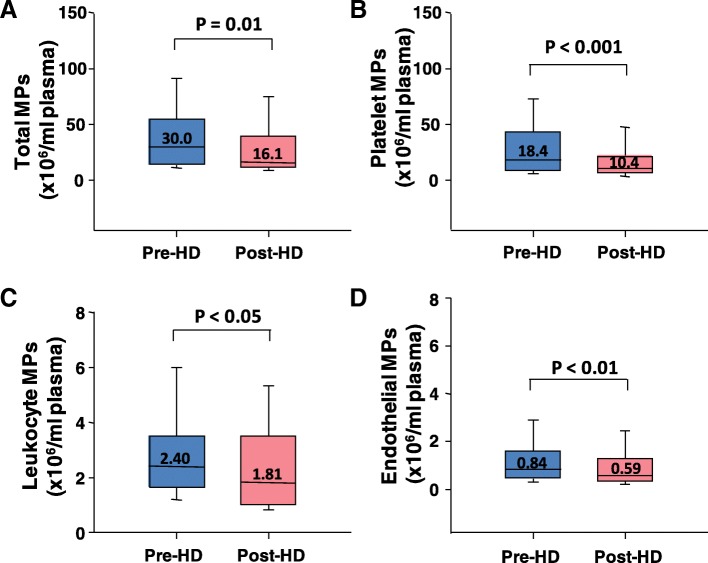
Quantitation of total (**a**), platelet (**b**), leukocyte (**c**), and endothelial (**d**) microparticle levels in plasma before (pre) and after (post) hemodialysis (HD)

To further assess the effect of hemodialysis on circulating EV levels, we performed flow cytometry analysis of MPs in plasma samples collected pre and post-dialysis. MPs from endothelial cells, leukocytes, and platelets as well as total MPs were all significantly reduced post-dialysis (Fig. 3).

### Relationship between circulating microparticles and ultrafiltration rate

One possible explanation for the reduction in MPs is that particles are filtered during dialysis. If this were the case, then we might expect that the amount of MPs cleared to be proportional to ultrafiltration rate. As shown in Table 3**,** levels of endothelial, leukocyte, platelet, and total MPs removed during dialysis did not correlate with ultrafiltration rate.

**Table 3:** Relationship between clearance of MP subpopulations and ultrafiltration rate

Microparticles	Spearman’s Correlation Coefficient	*p* value
Total (Annexin V +)	−0.306	NS
Platelet (CD41+)	−0.310	NS
Leukocyte (CD45+)	−0.322	NS
Endothelial (CD144+)	−0.385	NS

### Assessment of vesicle protein in dialysate and vesicle adsorption to the dialysis membrane

**Fig. 4:**
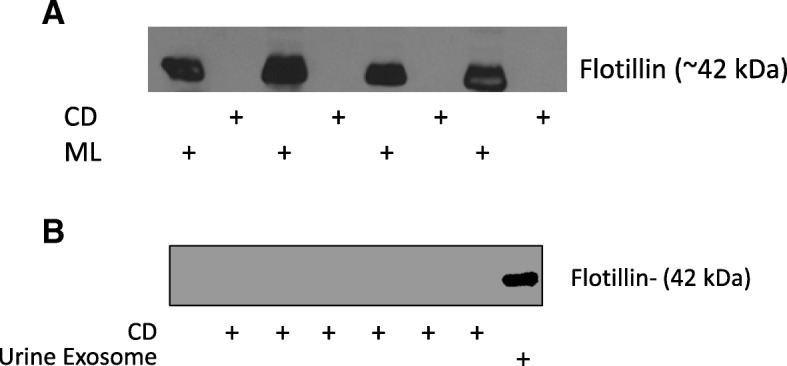
**a** Western blot analysis of concentrated dialysate (CD) and dialysis membrane lysate (ML) at end of dialysis session (4h). The vesicle-associated protein flotillin was detectable only in ML samples. **b**: Western blot analysis of CD at 1 h showing no detectable protein. Urine exosome isolates are included as positive control

To determine if EVs are filtered through or if they adsorb to the dialysis membrane we examined levels of the vesicle-associated protein flotillin by immunoblot. Flotillin was selected due to its high levels in all types of extracellular vesicles. Flotillin was not observed in concentrated dialysate samples at 1 or 4 h (Fig. 4). Similarly, levels of submicron particles in dialysate were not significantly different from background by nanoparticle tracking analysis (data not shown). By contrast, we observed the presence of flotillin in preparations from the dialysis membrane (Fig. 4) suggesting that EVs adsorb to the membrane.

## Discussion

The purpose of the present study was to examine the effect of hemodialysis on circulating extracellular vesicles and submicron particles. The principal finding is that hemodialysis reduced circulating levels of submicron particles including extracellular vesicles arising from endothelial cells, platelets, and leukocytes. The reduction in levels of vesicles does not appear to be due to ultrafiltration of particles but rather to adsorption of vesicles to the dialysis membrane. Taken together these results suggest that hemodialysis reduces levels of circulating submicron particles which may lead to significant interdialytic variation in patients with ESKD.

In individuals with ESKD, hemodialysis is the most common renal replacement therapy. Although it efficiently removes small molecular weight molecules, other constituents of plasma (protein-bound molecules, large molecular weight compounds) are not dialyzed to a significant degree. Moreover, hemodialysis may induce oxidative and/or hemodynamic stress leading to vascular injury, conditions which may increase formation of MPs. Indeed, a number of reports have suggested that circulating MPs are increased with hemodialysis [4, 5, 12]. However, it should be noted that amongst these studies, the specific subpopulations of MPs which are altered by hemodialysis varies. For example, Faure et al. observed increases in platelet MPs, but no change in endothelial or leukocyte MPs [5] whereas Daniel observed an increase in neutrophil, but not platelet MPs [4]. Our results show that all populations of circulating MPs measured (endothelial, leukocyte, platelet and total) are reduced by hemodialysis. These results are in agreement with Georgatzakou and colleagues who observed reductions in total and red blood cell-derived MPs post-dialysis [14]. In addition, using nanoparticle tracking analysis, we observed reductions in all particles 20–1000 nm in size. To the best of our knowledge, this is the first examination of the effect of hemodialysis on submicron particles to employ nanoparticle tracking analysis. Notably, nanoparticle tracking analysis assesses not only extracellular vesicles, but also other particles of similar size such as lipoproteins, protein aggregates, and viral particles [22]. Therefore, while consistent with our flow cytometry data of large EVs, the nanoparticle tracking data may be indicative of reductions in circulating levels of other, non-vesicular particles. Consistent with this, Dautin et al. have previously reported that circulating lipoprotein levels are reduced by hemodialysis [23]. It should also be noted that our observations apply specifically to hemodialysis and may not be applicable to other forms of extracorporeal circulation such as cardiopulmonary bypass. Indeed, there is evidence that this latter process is associated with increased levels of extracellular vesicles (reviewed in [24]).

Given the clear differences in submicron particles pre- and post-dialysis, we sought to determine the mechanism responsible for reduction. One possibility is that the particles are simply being filtered as is the case for small molecular weight molecules. We believe that this is highly unlikely for several reasons. First, the pore size on a dialysis filter (typically < 20 kDa) is far smaller than the diameter of even the smallest particles detected by nanoparticle tracking/flow cytometry. Second, we did not observe any correlation between ultrafiltration rate and the clearance of circulating MPs. Finally, when probing for the vesicle-associated protein flotillin, we were unable to detect any protein in concentrated dialysate samples. While it is possible that a certain amount of protein is present in dialysate but undetectable, we believe that the more likely explanation is that the vesicles are being removed by other means.

In this regard, we also sought to determine if vesicles adsorb to the dialysis membrane. Previous studies have reported significant adsorption of proteins and lipoproteins to dialysis membrane [23, 25]. Indeed, the removal of circulating inflammatory mediators by continuous renal replacement therapy is believed to be mediated, at least in part, via membrane adsorption [26]. Similarly, we observed the presence of flotillin on each of the dialysis membranes tested. Our interpretation is that vesicles are adsorbing to the dialysis membrane and that this is the most likely explanation for the reduced levels of circulating MPs post-dialysis. It is notable that Vergauwen et al. recently provided evidence of binding of EVs to cellulose and polysulfone-based centrifugal membranes [27]. Nevertheless, we cannot exclude the possibility that the flotillin we detected on the dialysis membrane was freely circulating rather than in vesicles when it adsorbed.

In summary, we observed a reduction in circulating submicron particles following hemodialysis. The reductions were observed for all subpopulations of particles studied, regardless of size or cellular origin. The reductions in particles/EVs was not associated with the appearance of vesicle protein in the dialysate and did not correlate with ultrafiltration rates suggesting that the particles are not filtered. Preliminary evidence suggests that particles adsorb to the dialysis membrane.

## Conclusions

The observation that levels of submicron particles are altered by dialysis has practical implications for the study of EVs in patients with ESKD. Investigators interested in measuring EVs in patients on hemodialysis should ensure that the timing of biosampling is coordinated so as to minimize the impact of interdialytic variation in circulating EVs. From a clinical standpoint, the fluctuation associated with dialytic removal and interdialytic gain in circulating EVs may lead to periods of increased hemodynamic stress since certain EV populations have been shown to induce vascular injury [21, 28, 29].

## Data Availability

The datasets used and/or analysed during the current study are available from the corresponding authors on reasonable request.

## Abbreviations

ESKD: End stage kidney disease
EV: Extracellular vesicle
MP: Microparticle
NTA: Nanoparticle tracking analysis
RIPA: Radio immune-precipitation assay
